# Human papillomavirus–related condyloma on keratinized skin

**DOI:** 10.1016/j.jdcr.2022.08.007

**Published:** 2022-08-11

**Authors:** Gyohei Egawa, Kenji Kabashima

**Affiliations:** Department of Dermatology, Kyoto University Hospital, Kyoto, Japan

**Keywords:** condyloma, dermoscopy, HPV, human papillomavirus, keratosis, seborrheic wart

## Clinical presentation

**Fig 1:**
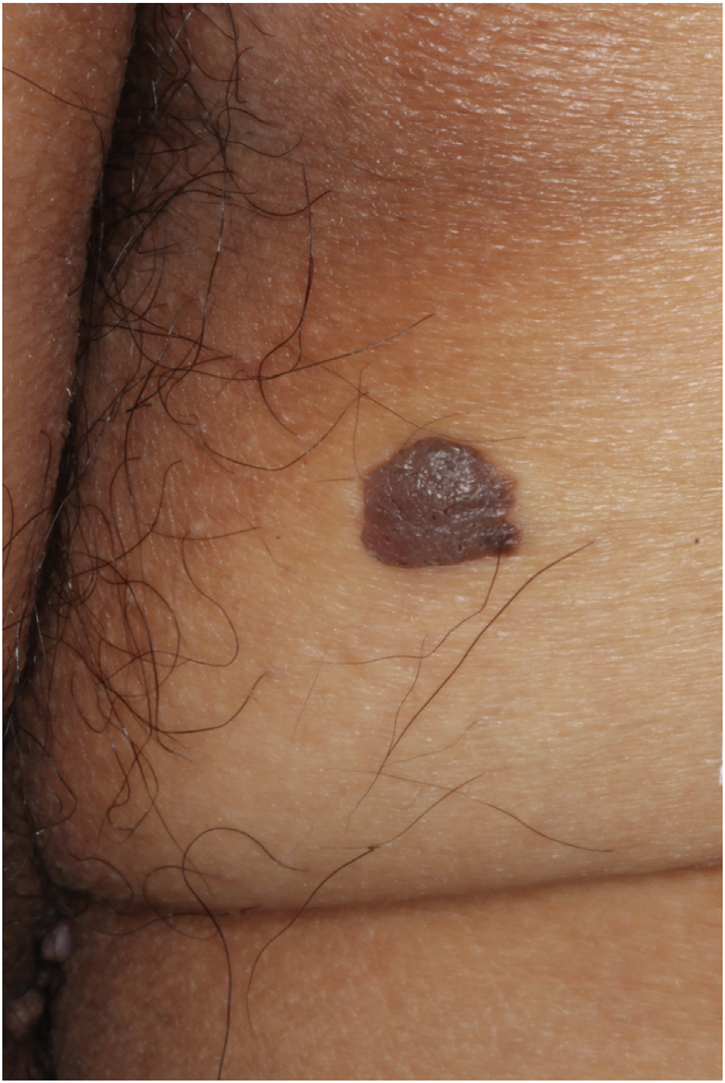
Clinical image of the lesion. The lesion was 15 mm in diameter, round, slightly elevated, brownish plaque with granular appearance, which resembled seborrheic keratosis.

A 72-year-old man presented with a brownish plaque on his right buttock (Fig 1). The plaque was 15 mm in diameter, round, slightly elevated, and granular, resembling seborrheic keratosis (SK). He had been receiving prednisolone 5 mg/day and cyclosporine 100 mg/day for 6 years for hypersensitivity pneumonitis.

## Dermoscopic appearance

**Fig 2:**
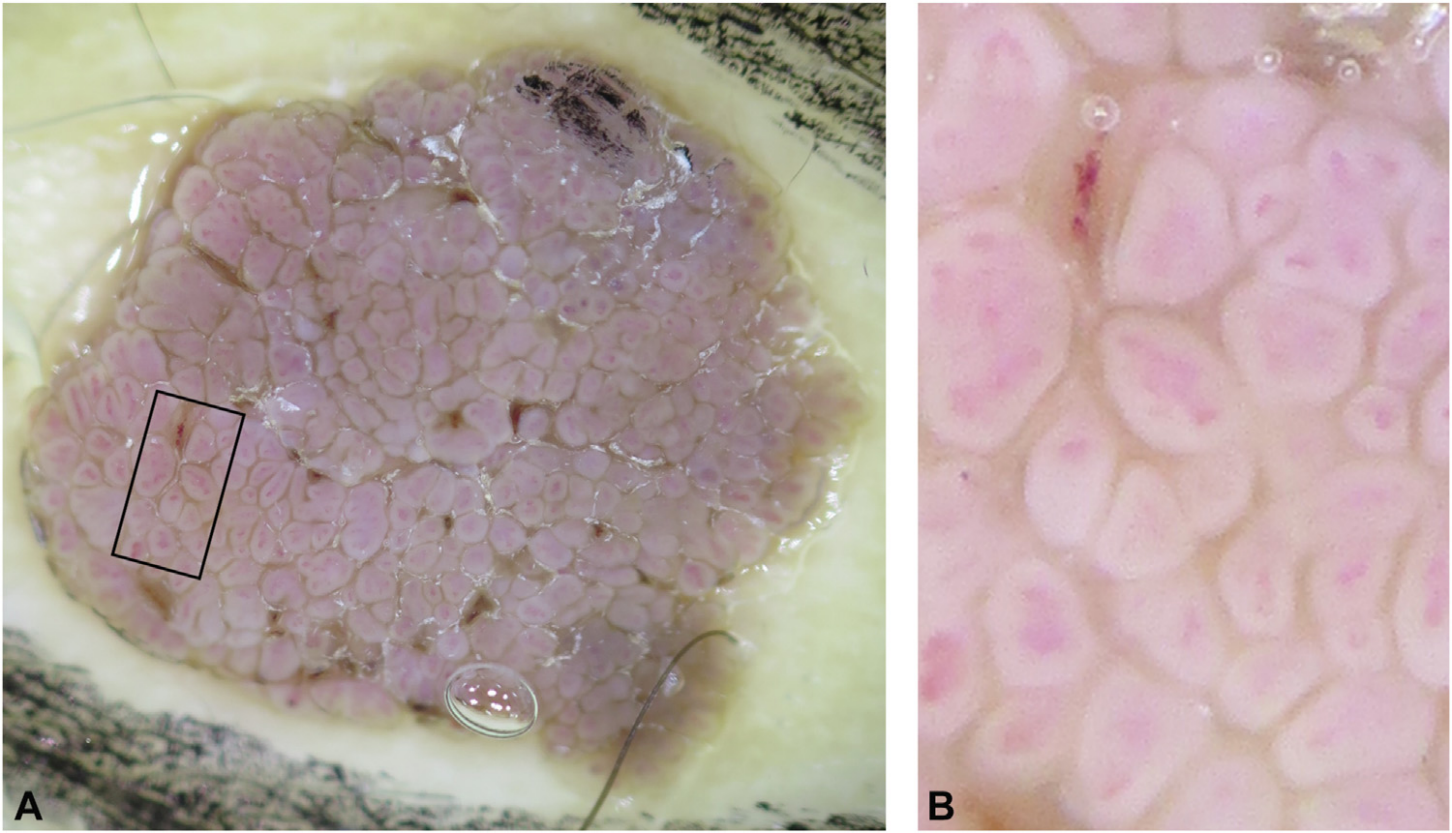
Dermoscopic images. **B,** shows the high magnification view of the *black square* area in (**A**). A slightly elevated *brownish* plaque with a papillary (frogspawn) pattern (**A**) and dotted and hairpin-like blood vessels in each papilla (**B**) were observed.

Dermoscopy showed a brownish plaque with a frogspawn pattern (Fig 2, *A*) and dotted hairpin-like blood vessels in each papilla (Fig 2, *B*).

## Histologic diagnosis

**Fig 3:**
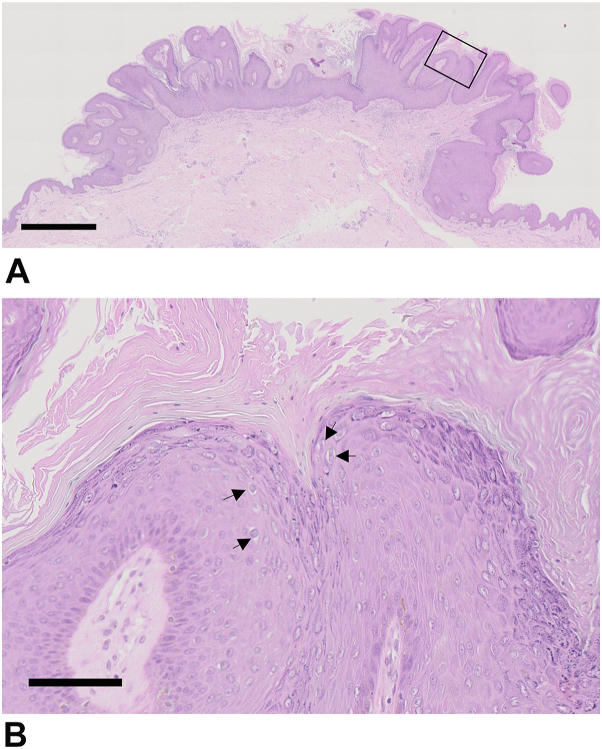
Hematoxylin and eosin staining of the lesion. **B,** shows the high magnification view of the *black square* area in (**A**). Papillary acanthosis with hyperkeratosis (**A**), parakeratosis, hypergranulosis, and a small number of koilocytes were observed (**B**, *arrows*). *Scale bars* are 1 mm in (**A**) and 100 μm in (**B**).

Papillary acanthosis with hyperkeratosis (Fig 3, *A*), parakeratosis, hypergranulosis, and a few koilocytes (Fig 3, *B*, arrows) were observed. Human papillomavirus (HPV)–6 was detected by polymerase chain reaction.

**Key message**

Condyloma acuminatum, a common sexually transmitted disease caused by low-risk HPVs (HPV-6 or 11), usually occurs on the anogenital mucosa but may also occur on keratinized skin, especially in immunocompromised hosts. On keratinized skin, HPV-6/11 form flat brownish plaques, which sometimes mimic SK; however, they are distinguishable by dermoscopy.^1^ Comedo-like openings and milia-like cysts found in 71% and 66% of SK, respectively,^2^ are absent in condyloma. While vascular structures are rarely observed in SK, dilated blood vessels are observed in most cases of condyloma. The characteristic dermoscopic findings of condyloma are still observed even on keratinized skin, although the clinical picture differs from condyloma acuminatum on the mucosa. Dermatologists should be familiar with these clinical and dermoscopic pictures of condyloma on keratinized skin because of the need for appropriate treatment, such as topical imiquimod and cryotherapy, and patient education to prevent HPV infection.

## Conflicts of interest

None disclosed.
